# The importance of *Calanus glacialis* for the feeding success of young polar cod: a circumpolar synthesis

**DOI:** 10.1007/s00300-020-02643-0

**Published:** 2020-03-11

**Authors:** Caroline Bouchard, Louis Fortier

**Affiliations:** 1 Greenland Climate Research Centre, Greenland Institute of Natural Resources, 3900 Nuuk, Greenland; 2 Québec-Océan, Département de Biologie, Université Laval, Québec, QC G1V 0A6 Canada

**Keywords:** Arctic cod, *Boreogadus saida*, *Calanus glacialis*, Borealization, Fish larvae, Age-0 fish

## Abstract

Understanding the feeding ecology of polar cod (*Boreogadus saida*) during its first year of life is crucial to forecasting its response to the ongoing borealization of Arctic seas. We investigated the relationships between diet composition and feeding success in 1797 polar cod larvae and juveniles 4.5–55.6 mm standard length (SL) collected in five Arctic seas from 1993 to 2009. Prey were identified to species and developmental stages when possible, measured, and their carbon content was estimated using taxon-specific allometric equations. Feeding success was defined as the ratio of ingested carbon to fish weight. Carbon uptake in polar cod larvae < 15 mm was sourced primarily from calanoid copepods eggs and nauplii which were positively selected from the plankton. With increasing length, carbon sources shifted from eggs and nauplii to the copepodites of *Calanus glacialis*, *Calanus hyperboreus* and *Pseudocalanus* spp. *Calanus glacialis* copepodites were the main carbon source in polar cod > 25 mm and the only copepodite positively selected for. *Pseudocalanus* spp. copepodites became important replacement prey when *C. glacialis* left the epipelagic layer at the end of summer. *Calanus glacialis* was the preferred prey of polar cod, contributing from 23 to 84% of carbon uptake at any stage in the early development. Feeding success was determined by the number of prey captured in larvae < 15 mm and by the size of prey in juveniles > 30 mm. As Arctic seas warm, the progressive displacement of *C. glacialis* by the smaller *Calanus finmarchicus* could accelerate the replacement of polar cod, the dominant Arctic forage fish, by boreal species.

## Introduction

Thanks to large biomasses and a high degree of trophic connectivity, polar cod (*Boreogadus saida*) plays a pivotal role in Arctic marine ecosystems. Alterations of its ecology and abundance by climate change may cascade in the pelagic food web and directly affect the many services provided to local communities by the predatory fish, marine mammals and seabirds feeding on polar cod (Welch et al. 1992; Tynan and DeMaster 1997; Darnis et al. 2012). Recent studies have linked interannual and regional variations in the recruitment of juvenile polar cod to the date of the ice break-up, which dictates the overall production of the ecosystem during the first weeks or months of planktonic life of the epipelagic larvae and juveniles (Bouchard et al. 2017; LeBlanc et al. 2019). Deciphering the precise mechanism(s) linking early survival to ecosystem production is a basic requirement towards modeling and forecasting the response of polar cod to the ongoing borealization of Arctic seas.

Fish larvae and newly-metamorphosed juveniles often select one or a few specific prey taxa in the plankton (e.g., Dickmann et al. 2007; Robert et al. 2008; Llopiz and Cowen 2009; Young et al. 2010; Murphy et al. 2012). Robert et al. (2013) emphasized the importance of identifying the preferred prey at the lowest taxonomical level possible to understand the trophodynamics of young fish and their response to a fluctuating or changing plankton environment. Polar cod larvae and juveniles feed primarily on the eggs, nauplii and copepodites of a few copepod species including the large, lipid-rich *C. glacialis* and *C. hyperboreus* endemic to the High Arctic (Bouchard et al. 2016). Climate warming is altering the distribution of Arctic zooplankton in ways that could negatively affect the feeding success of young polar cod. For instance, a general decrease in the proportion of large zooplankton in Arctic seas (e.g., Balazy et al. 2018; Møller and Nielsen 2019) and the northward penetration of the smaller *C. finmarchicus* (e.g., Beaugrand et al. 2013) could reduce the availability of high-energy prey to polar cod larvae and juveniles.

In this meta-analysis, we assemble gut content data sets for polar cod 4.5–55.6 mm long sampled in different years in five Arctic seas (total of seven year-sea combinations) to build a composite picture of the diet (in terms of carbon uptake) of the species during its early ontogeny and to identify its preferred prey at the lowest taxonomic level possible. Diet and feeding success (gut content carbon/fish weight) are contrasted among the Beaufort and Greenland Seas, Baffin Bay and the Kara-Laptev Seas area. The importance of prey number and prey size in determining regional differences in feeding success is assessed.

## Materials and methods

### Study areas

**Fig. 1:**
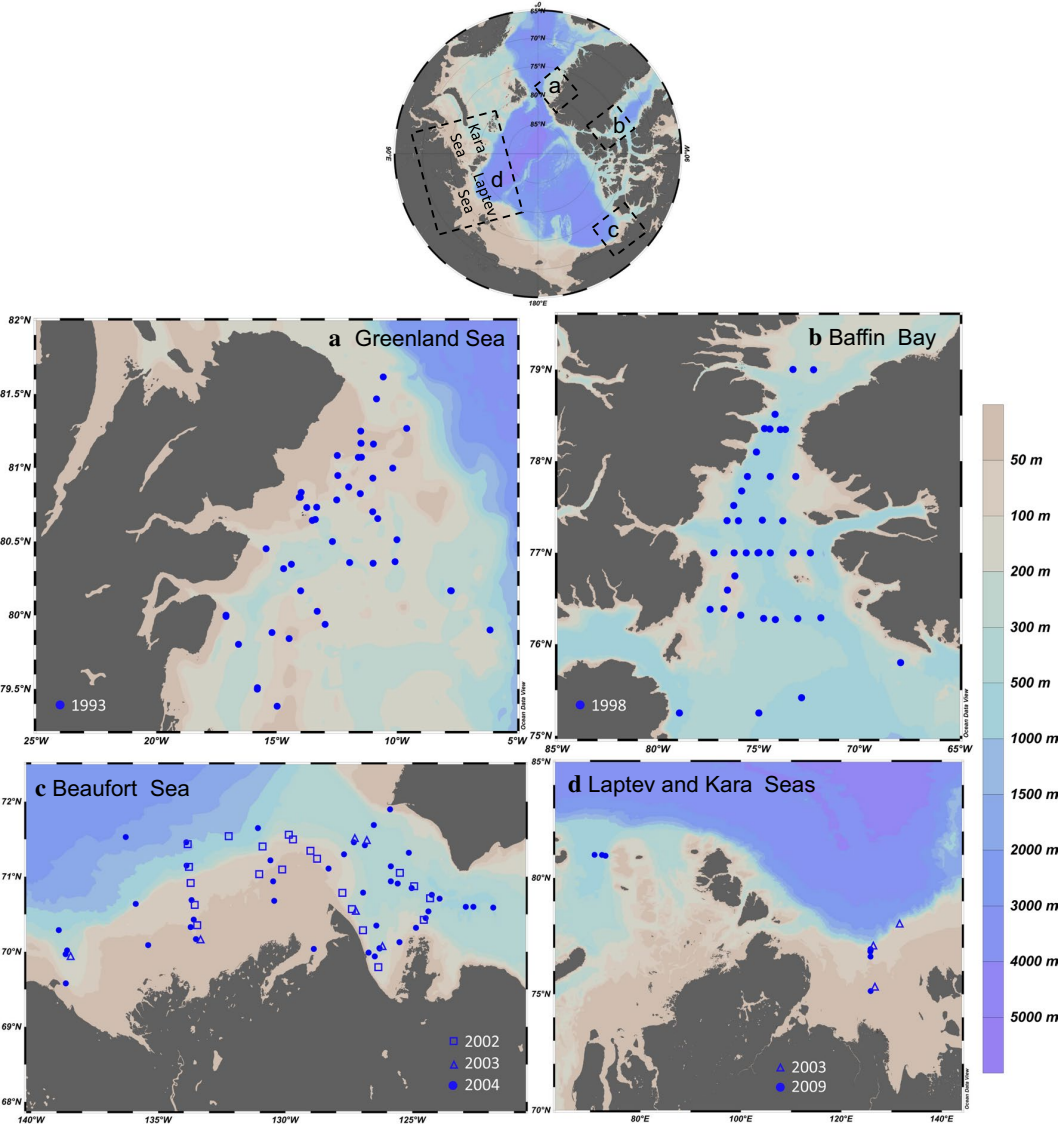
Bathymetric map of the Arctic Ocean indicating the four regions studied (top). Panels **a**–**d** presents the bathymetric maps of the different seas sampled with symbols indicating the location and years of sampling of polar cod larvae and juveniles

Polar cod larvae and epipelagic juveniles were collected in five Arctic seas at stations distributed on the continental shelves and slopes in the depth range 31–3000 m (Fig. 1). The sampling was part of four international research programs between 1993 and 2009, resulting in 7 year-sea combinations (Table 1). Given that only three stations were sampled in the Kara Sea in the single year 2009, the Laptev Sea and Kara Sea collections for 2009 were grouped into a single year-sea combination henceforth referred to as the Laptev 2009 combination. The combination appears justified since the stations in these seas are located in areas with similar oceanographic conditions in terms of Atlantic water input, freshwater influence, sea-surface temperature, and sea-ice dynamics (Xiao et al. 2013 and references therein). Briefly, the Beaufort Sea on the North-American side of the Arctic Basin is influenced by the Mackenzie River in summer and the Cape Bathurst polynya in winter (Carmack and MacDonald 2002 and references therein). The Laptev and Kara seas on the Siberian side are influenced by the Lena River and the Ob and Yenisei rivers respectively and by winter polynyas (Zakharov 1997). The Northeast Water in the Greenland Sea and the North Water in Northern Baffin Bay are two large polynyas with little freshwater influence (Barber and Massom 2007). Except for the Greenland Sea where Atlantic forms can be common (Ashjian et al. 1997; Hirche and Kwasniewski 1997), the zooplankton assemblages of the sampled seas are typically Arctic with few Atlantic or Pacific expatriates (e.g., Kosobokova et al. 1998; Ringuette et al. 2002; Darnis et al. 2008).

**Table 1:** Number of larval and juvenile polar cod gut content analyzed by year-sea combinations, with capture periods and standard length (SL) range

Sea	Program	Dates and years	SL (mm)	Number
Greenland Sea	NEW	23 May–02 Aug 1993	6.5–23.2	530
North Baffin Bay	NOW	29 Apr–20 Jul 1998	4.5–16.3	814
Beaufort Sea	CASES	22 Sep–14 Oct 2002	15.8–45.0	138
		30 Sep–28 Dec 2003	25.0–51.4	30
		15 May–12 Sep 2004	5.7–54.0	193
Laptev Sea	NABOS	03–11 Sep 2003	17.1–55.6	64
Laptev Sea (and Kara Sea)		23–28 Aug 2009	15.0–32.0	28

*NEW* Northeast Water Polynya Program, *NOW* International North Water Polynya Program, *CASES* Canadian Arctic Shelf Exchange Study, *NABOS* Nansen and Amundsen Basins Observational System

### Ichthyoplankton and zooplankton sampling

The sampling methodology is detailed in previous publications (Michaud et al. 1996; Fortier et al. 2006; Thanassekos and Fortier 2012; Bouchard et al. 2016). In summary, zooplankton and fish larvae were collected with a bongo (Greenland Sea) or a double square net sampler (Beaufort Sea, Laptev Sea and Baffin Bay) carrying nets with mesh sizes ranging from 200 to 750 µm (depending on sampling month), and an additional 10-cm diameter cylindrical net with 50- or 64-μm mesh to sample small polar cod prey such as copepod eggs and nauplii. The samplers were deployed at 2–3 knots in simple or double oblique tows from the surface to between 50 and 100 m. The zooplankton assemblage, integrated over the entire water column, was also sampled with vertical samplers carrying 200-μm mesh and 50-μm nets. Fish larvae and juveniles were sorted from zooplankton samples, measured fresh (standard length) and individually preserved in 95% ethanol at sea.

### Gut content analysis and prey identification

The digestive tracts of 1797 polar cod were dissected in glycerol and 74,498 prey were examined under the stereomicroscope by 11 different taxonomists all trained in the same laboratory at Université Laval (Quebec City, Canada). Prey were identified to the lowest taxonomic level possible and classified in the following most abundant taxa used in the analyses: copepod eggs, cyclopoid nauplii, *Pseudocalanus* spp. nauplii, *Calanus* spp. nauplii, unidentified nauplii; copepodite stages of *C. glacialis*, *C. hyperboreus*, *Pseudocalanus* spp., and *Oithona similis*; unidentified copepods; and others.

The category “Others” included in order of decreasing frequency: bivalve larvae, appendicularians, gastropod larvae, *Paraeuchaeta* spp., *Metridia* nauplii, unidentifiable digested material, *Microcalanus* spp., *Oncaea parila*, cladocerans, *Limnocalanus* spp., *C. finmarchicus* copepodites, harpacticoid copepods, *Microcalanus* nauplii, tintinnids, *Eurytemora* nauplii, *Metridia longa*, *Acartia* spp., *Paraeuchaeta* nauplii, chaetognaths, cnidarians, polychaetes, amphipods, branchiopods, *Acartia* nauplii, *Spinocalanus* spp, rotifers, *Neomormollida* spp., cirripeds, *C. pacificus*, ostracods, *Scolecithrella minor*, *Triconia minuta*, isopods, and echinoderm larvae.

All recognizable prey items were measured: diameter for copepod eggs; prosome length for copepod nauplii and copepodites; maximum length for other taxa. Copepod eggs were assigned to species based on their diameter (Brun et al. 2017 and references therein; Michaud et al. 1996; Daase et al. 2011 and references therein) using the following classification: 40–60 μm: *Triconia borealis*, 60–80 μm: *Oithona similis*, 80–100 μm: *Microcalanus* spp., 100–150 μm: *Pseudocalanus* spp., 150–170 μm: *Metridia longa*, 170–210 μm: *C. glacialis*, < 40 μm and > 210 μm: other. Since *C. hyperboreus* spawn in winter, their eggs were probably not available for the young polar cod sampled in spring–summer (at the earliest in mid-May; Table 1). Hence no eggs were assigned to this species. Copepod nauplii and copepodites were classified into developmental stages (N1 to N6 and C1 to C5, adult female, adult male) when possible.

Hirche and Kwasniewski (1997) established that in the Greenland Sea, *C. finmarchicus* does not spawn from May to the end of July and probably not at all. Hence the copepod eggs and *Calanus* spp. nauplii prey of polar cod sampled from May to early August in the Greenland Sea were unlikely to belong to *C. finmarchicus*. *Calanus* spp. nauplii, which cannot be discriminated based on morphometrics and size in preserved samples (Melle and Skjoldal 1998; Jung-Madsen and Nielsen 2015), could either be *C. glacialis* or *C. hyperboreus*. However, in the Beaufort Sea, for instance, a first cohort of *Calanus* spp. nauplii occurs from February to mid-March (Ota et al. 2008; Daase et al. 2013). These winter nauplii are most likely *C. hyperboreus* whose spawning takes place in early winter and is completed by early April (Ota et al. 2008; Darnis et al. 2012; Daase et al. 2013). Hence, following Ota et al. (2008) and Daase et al. (2013), the *Calanus* spp. nauplii prey in polar cod sampled in late spring and summer were assigned to *C. glacialis* in the calculation of the contribution of calanoid copepods to the carbon uptake of polar cod larvae.

The reliability of morphometric criteria in discriminating *C. glacialis* and *C. finmarchicus* copepodites has been questioned (e.g., Lindeque et al. 2006; Parent et al. 2011; Gabrielsen et al. 2012; Choquet et al. 2017). Based on molecular identification, *C. finmarchicus* is absent from the Beaufort Sea and northern Baffin Bay (e.g., Choquet et al. 2017; Parent et al. 2011), thus *C. glacialis* copepodite prey were almost certainly correctly identified for these seas. The possibility exists that some of the copepodites identified as *C. glacialis* in the gut content of polar cod sampled in the Greenland, Kara and Laptev seas were actually expatriate *C. finmarchicus* (Choquet et al. 2017). However, differences in the prosome length of the copepodites of the two species are greatest at high latitudes in the northern part of their co-distribution (Hirche 1991; Parent et al. 2011; Abyzova and Stupnikova 2017), and the identification of *C. glacialis* copepodites in the gut of Greenland Sea polar cod based on prosome length is probably correct. Due to their scarcity, the few (*n* = 4) *C. finmarchicus* identified in the gut of polar cod from the Greenland Sea ended up in the “Others” category.

The carbon content of each prey was estimated using published carbon-length, length–weight and carbon-weight relationships following Bouchard et al. (2016). For each polar cod, a weight-independent feeding success was calculated by dividing ingested carbon by the weight of the larva or juvenile, estimated as *W* = 0.0055 (SL)^3.19^ where SL is the fresh standard length of the fish (Geoffroy et al. 2016).

Assuming that *Calanus* spp. nauplii prey were *C. glacialis* (see above for rationale), a composite (all years-seas) picture of the overall contribution of each of the three main copepod prey (*Pseudocalanus* spp., *C. glacialis* and *C. hyperboreus*) was built by summing the estimated contribution of the eggs, nauplii, and copepodites of each taxon to the carbon intake of polar cod of different lengths.

### Selectivity for prey

Detailed analysis of the microzooplankton samples from the 50-µm mesh nets was available for the shallow Laptev Sea (2003, bottom to surface vertical tows) and the Beaufort Sea (2004, epipelagic oblique tows). A subset of two stations in the Laptev Sea (depths of 100 m and 200 m) and two stations in the Beaufort Sea (depths of 41 m and 225 m) where young polar cod were abundant was used to determine the selectivity of polar cod larvae and juveniles for their prey using Chesson’s *α* index (Chesson 1978):

$$\alpha_{j } = \frac{{\left( {\frac{{d_{j } }}{{p_{j } }}} \right)}}{{\sum {\left( {\frac{{d_{i} }}{{p_{i } }}} \right)} }}\quad {\text{for}}\,i\, = \,1 \ldots N$$

where *N* is the number of prey taxa considered; *d*_*j*_/*p*_*j*_ is the ratio of the relative frequencies of prey *j* in the diet (*d*_*j*_) and in the plankton (*p*_*j*_); and Σ(*d*_*i*_/*p*_*i*_) the sum of this ratio over all prey taxa. For each station, successive known aliquots of the microzooplankton net sample were analyzed until a minimum of 300 organisms were identified. For the Laptev Sea, large copepodite and adult stages were enumerated in the 200-µm mesh net collections due to potential avoidance of the 50-µm mesh nets. For the Beaufort Sea, all taxa were enumerated from the 50-µm mesh net oblique tows since the largest prey found in the gut contents (*Pseudocalanus* spp. female and *C. glacialis* C1) were small enough to be adequately quantified by this mesh size. For a length category of larvae/juvenile, only taxa representing > 1% of the overall carbon uptake by polar cod were included in the calculation of the selectivity index. Copepodite stages were considered as separate taxa in the calculation of α. Prey selectivity was computed for each fish and then averaged for two length classes (< 25 mm, > 25 mm).

### Statistics

Mean prey size, mean number of prey and mean feeding success within each 1-mm (fish < 25 mm SL) and 2-mm (fish > 25 mm SL) length interval of polar cod were compared among seas with one-way ANOVAs, followed by pairwise comparisons using Tukey HSD tests. A negative binomial regression was used to model the relationship between the number of *C. glacialis* copepodites ingested and the standard length of young polar cod. The significance level of all statistical test was set at *p* value < 0.05.

## Results

### The general diet of young polar cod across seas

**Fig. 2:**
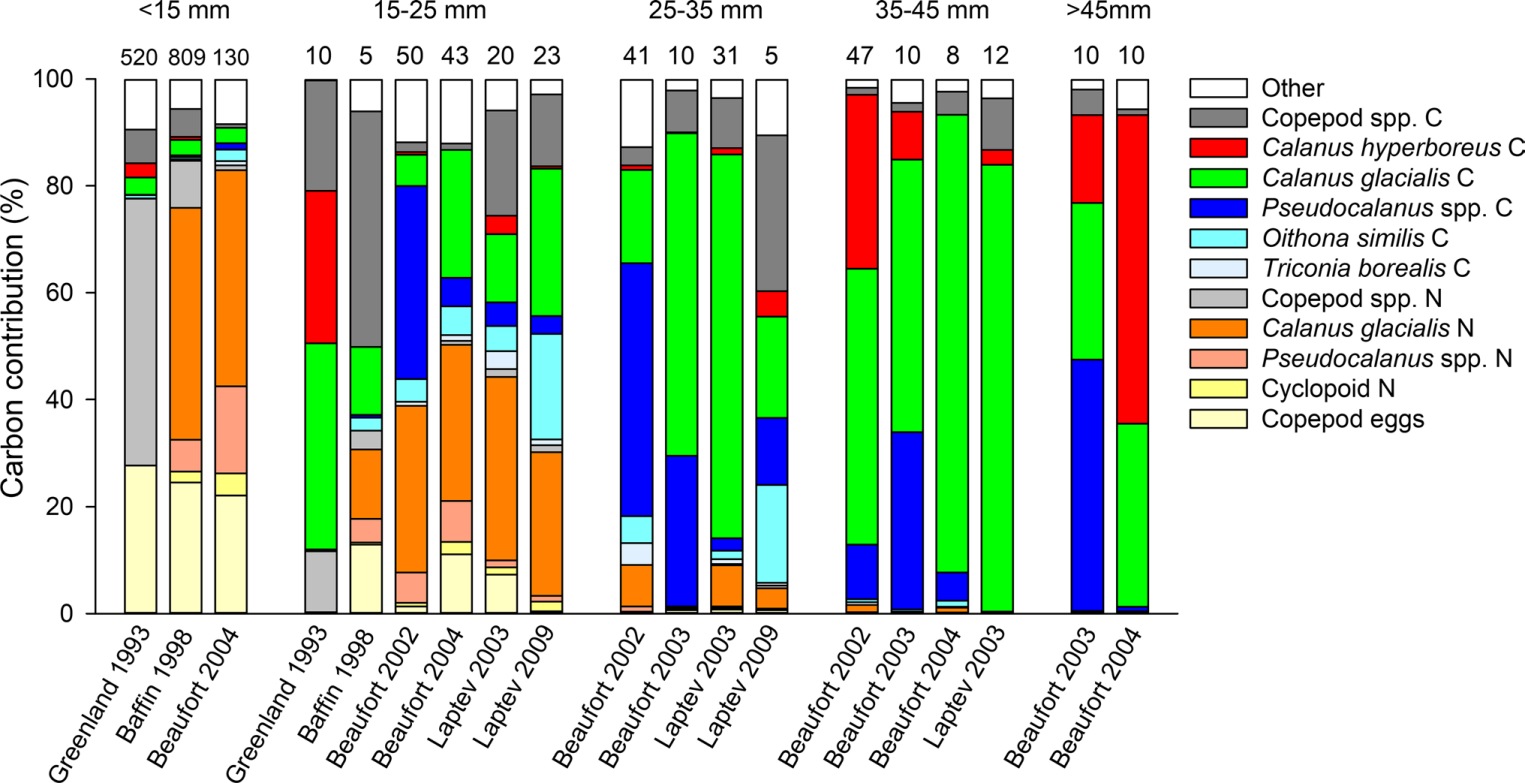
Relative contribution (%) of different prey to the carbon intake of different length classes of polar cod sampled between 1993 and 2009 in four Arctic seas. Numbers above histograms are the number of digestive tracts analyzed. Year-sea combinations for which < 5 tracts were analyzed are not presented. A given length class was not necessarily sampled in every year-sea combination. *N* nauplii, *C* copepodites

The diet of a given length class of polar cod was remarkably similar across year-sea combinations (Fig. 2). Assuming that *Calanus* spp. prey were *C. glacialis* (see Materials and Methods), larvae < 15 mm obtained most of their carbon from copepod eggs and the nauplii of *C. glacialis* and *Pseudocalanus* spp. Polar cod 15–25 mm also preyed on eggs and calanoid nauplii, and added the copepodites of *C. glacialis*, *Pseudocalanus* spp., *Oithona similis* and the infrequent *C. hyperboreus* as carbon sources. The carbon intake of polar cod 25–35 mm was sourced primarily from copepodites of *C. glacialis,* and *Pseudocalanus* spp., with minor contributions from *O. similis* and *C. hyperboreus* copepodites. *Calanus glacialis* copepodites were particularly important in the diet of juvenile polar cod 35–45 mm long. Carbon intake in juvenile polar cod 35–45 mm and > 45 mm came almost entirely from the copepodites of *Pseudocalanus* spp., *C. glacialis* and *C. hyperboreus*.

### Detailed diet: contribution of copepods to carbon uptake by species and stages

**Fig. 3:**
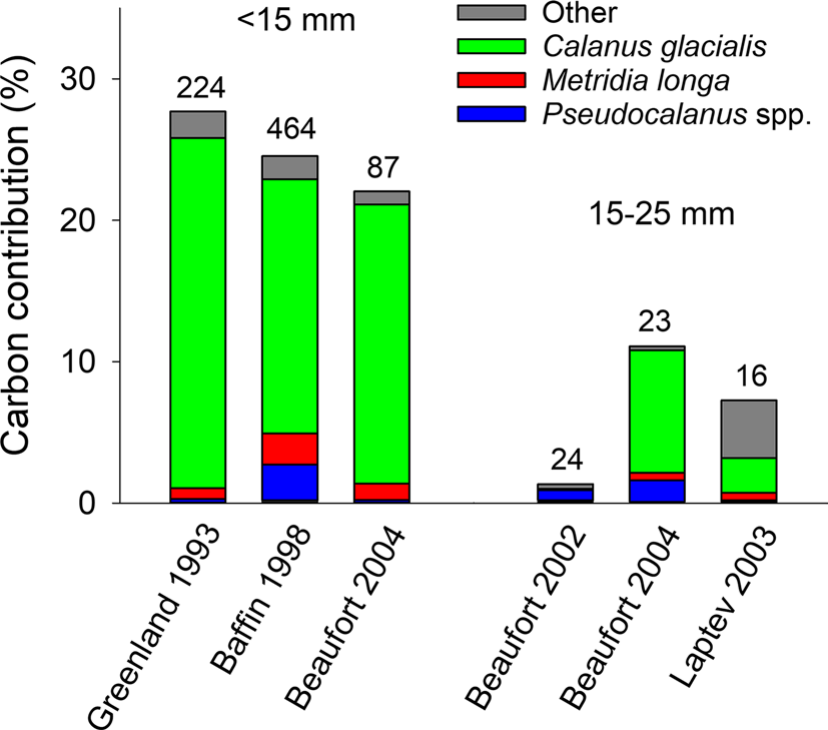
Contribution (%) of the eggs of copepod taxa to the carbon intake of different length classes of polar cod by year-sea combinations. Numbers above histograms are the number of digestive tracts analyzed. Year-sea combinations for which < 5 digestive tracts were analyzed or for which copepod eggs contributed to < 1% of the carbon intake are not presented. The very low carbon contribution of the eggs of *Triconia borealis*, *Oithona similis*, and *Microcalanus* spp. is included at the bottom of the histograms but barely visible

The relatively large eggs of *C. glacialis* contributed the bulk of total prey carbon sourced from copepod eggs (22.1–27.7%) by polar cod < 15 mm from all year-sea combinations (Fig. 3). The carbon fraction provided by copepod eggs was much less (1.3–11.0%) in larvae 15–25 mm and was contributed primarily by *Pseudocalanus* spp. in the Beaufort Sea in 2002, by *C. glacialis* in the Beaufort Sea in 2004, and by other species in the Laptev Sea in 2003 (Fig. 3). Copepod eggs contributed < 1% of the total carbon intake of polar cod > 25 mm.

**Fig. 4:**
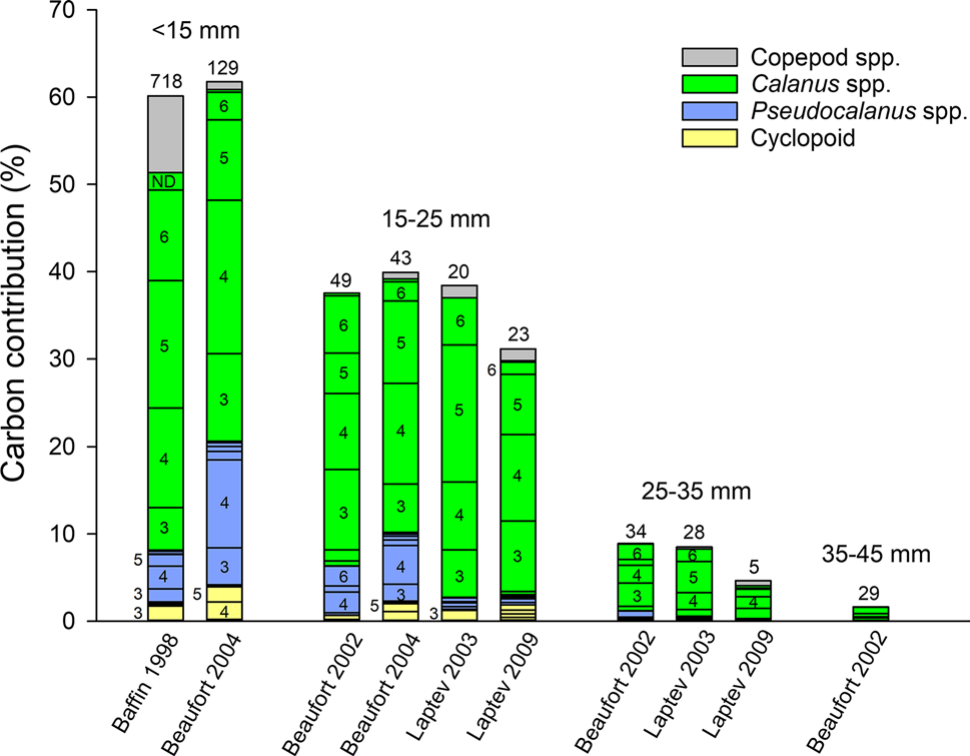
Contribution (%) of the naupliar stages of copepod taxa to the carbon intake of different length classes of polar cod by year-sea combinations. Numbers above histograms are the number of digestive tracts analyzed. Numbers and letters in stacked histograms correspond to naupliar stages (e.g., *5* N5). ND: non-determined naupliar stage of the taxon. Year-sea combinations for which < 5 digestive tracts were analyzed or for which copepod nauplii contributed to < 1% of the carbon intake are not presented. Greenland Sea 1993 is not presented since the nauplii were not classified into stages

The fraction of total carbon intake contributed by copepod nauplii was large (61%) in polar cod < 15 mm and declined rapidly with increasing length to < 1% in juveniles > 35 mm (Fig. 4). Polar cod from all year-sea combinations preyed primarily on the last non-feeding (N3) and the feeding naupliar stages (N4-N6) of the different species. Assuming that *Calanus* spp. nauplii were *C. glacialis* (see Materials and Methods), *C. glacialis* nauplii contributed most of the naupliar carbon in the diet of young polar cod (Fig. 4).

**Fig. 5:**
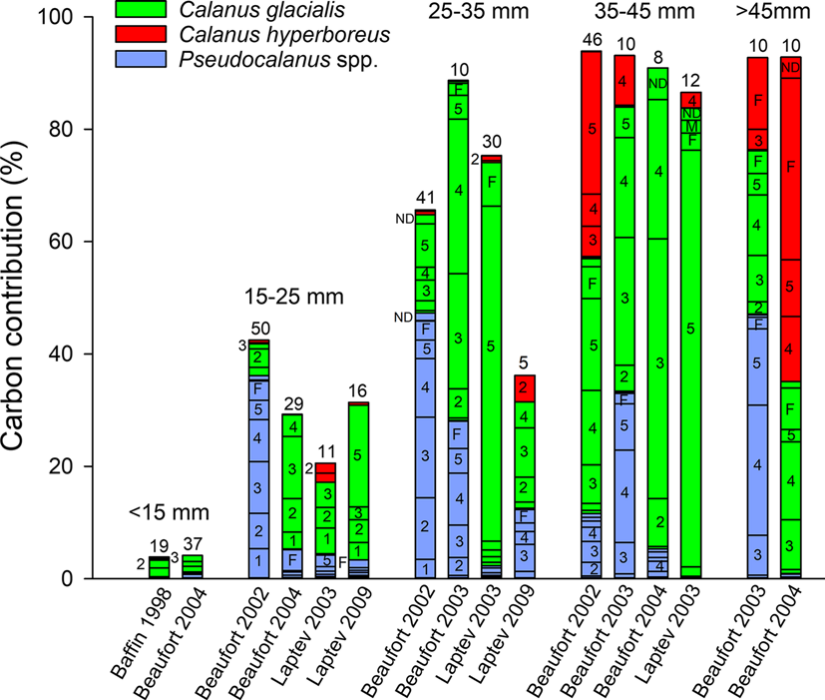
Contribution (%) of the copepodite stages of the three main copepodite prey (*Pseudocalanus* spp., *Calanus glacialis*, *Calanus hyperboreus*) to the carbon intake of different length classes of polar cod by year-sea combination. Numbers above histograms are the number of digestive tracts analyzed. Numbers and letters in stacked histograms correspond to copepodite stages (e.g., *1* C1, *F* female). ND: non-determined copepodite stage of the species. Year-sea combinations for which < 5 digestive tracts were analyzed are not presented. Greenland Sea 1993 is not presented since the copepodites were not classified into stages

The carbon sourced from the copepodites of the calanoids *Pseudocalanus* spp., *C. glacialis* and *C. hyperboreus* increased with polar cod length from less than 10% in larvae < 15 mm to over 90% in juveniles > 35 mm (Fig. 5). The contribution of *Pseudocalanus* spp. varied from 0 to nearly 45% among years-seas, being high in the Beaufort Sea in 2002 and 2003, but not in 2004, and always low in the Laptev Sea, with no clear trend as polar cod developed. When preying on this species, polar cod < 35 mm obtained carbon almost equally from all copepodite stages (C1 to F), while juveniles > 35 mm in the Beaufort Sea specialized on C3, C4 and C5.

As they increased in length, young polar cod preyed on increasingly large copepodites of *C. glacialis* (Fig. 5). C1–C3 *C. glacialis* were the main source of copepodite carbon for larvae < 25 mm, except in the Laptev Sea in 2009 where the large C5 were also important. The importance of C3, C4, C5, and F as carbon sources increased in polar cod > 25 mm. In the Laptev Sea in 2003, polar cod 25–45 mm sourced their copepod carbon almost exclusively from *C. glacialis* C5. In the Beaufort Sea in 2004, juveniles 35–45 mm obtained nearly all their carbon from C2–C4 *C. glacialis*. Polar cod < 35 mm infrequently captured the smaller copepodites of *C. hyperboreus* (C1 and C2) and this copepod contributed little to their carbon intake (Fig. 5). In the Beaufort Sea in 2002 and 2004, juveniles > 35 mm obtained a large fraction of their carbon from large C3 to F *C. hyperboreus*.

**Fig. 6:**
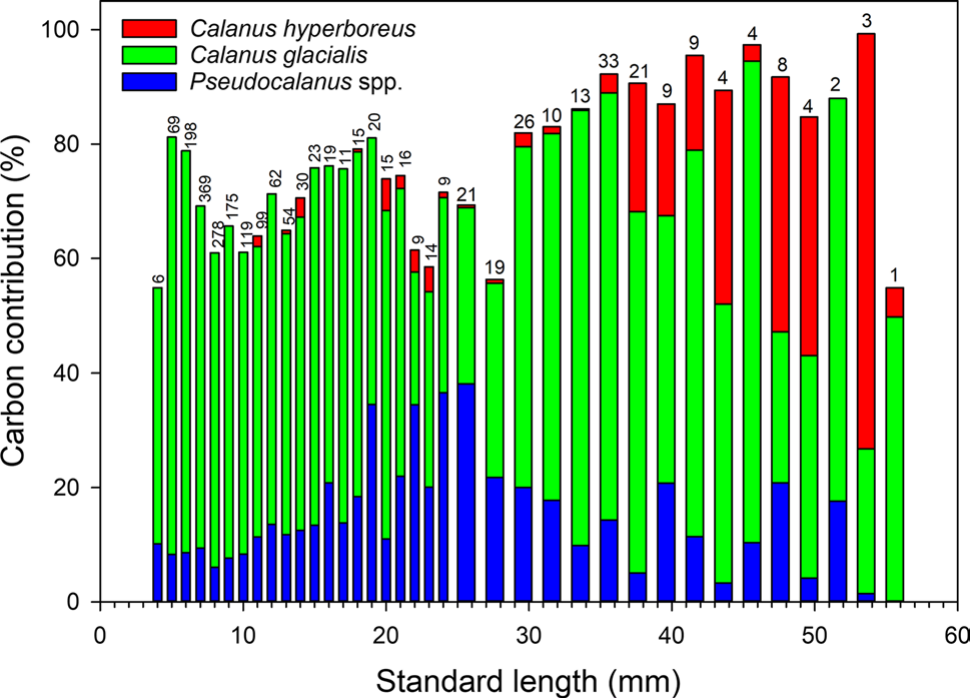
The carbon contribution of the three main copepod prey of polar cod summed over developmental stages (eggs + nauplii + copepodites) in relation to polar cod length. Bar width indicates the size of the length interval: 1 mm for larvae < 25 mm SL and 2 mm for individuals > 25 mm SL. *Calanus* spp. nauplii were assigned to *Calanus glacialis* (see Materials and Methods). Numbers above histograms are the number of digestive tracts analyzed

The contribution to the carbon intake of polar cod of the three main calanoid copepods (*Pseudocalanus* spp., *C. glacialis* and *C. hyperboreus*) summed over developmental stages (eggs + nauplii + copepodites) increased from about 70% in larvae and juveniles < 29 mm long to about 90% in juveniles > 29 mm (Fig. 6). Assuming that all *Calanus* spp. nauplii prey were *C. glacialis*, the summed contribution of *C. glacialis* dominated (23 to 84%) the carbon intake of young polar cod. *Calanus hyperboreus* copepodites became an increasingly important carbon source starting at 37 mm. The carbon contribution of *Pseudocalanus* spp. generally increased from < 10% in small larvae to 38% in larvae 25 mm long, and then varied between 0 and 22% in polar cod 25–50 mm.

### Prey selection by polar cod

**Fig. 7:**
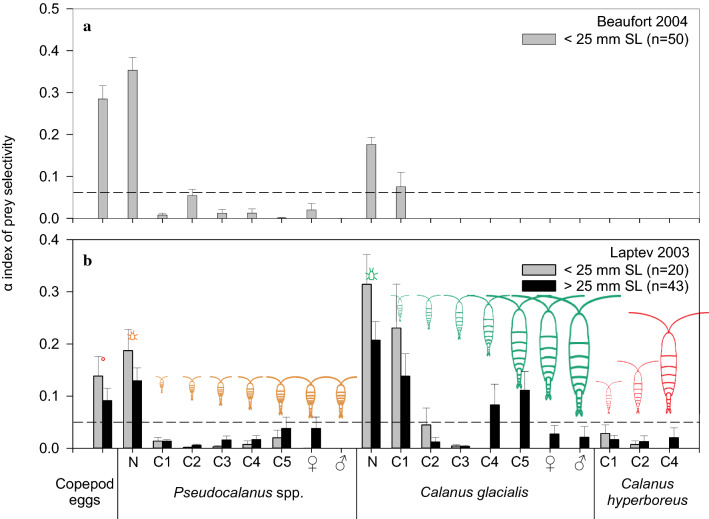
Chesson’s α index of prey selectivity for polar cod of two length classes. The horizontal line represents the 1/*n* prey taxa threshold where selectivity is neutral. Selectivity for a prey taxon is positive when α is higher than the threshold and negative otherwise. *N* nauplii, *C* copepodites. *Calanus* spp. nauplii were assigned to *Calanus glacialis* (see Materials and Methods). A pictogram above each histogram indicates the mean size of each prey taxon, from 0.142 mm for the mean diameter of copepod eggs to 3.26 mm for the mean prosome length of female *C. glacialis*. Vertical bars indicate standard errors

Polar cod larvae < 25 mm sampled in the Beaufort Sea in 2004 and the Laptev Sea in 2003 selected essentially the same prey (Fig. 7). Selectivity of polar cod > 25 mm was estimated in the Laptev Sea only. Both length classes positively selected copepod eggs, the nauplii of *Pseudocalanus* spp. and *C. glacialis* (assuming that *Calanus* spp. nauplii were *C. glacialis*) and the C1 copepodites of *C. glacialis*. Polar cod > 25 mm also selected *C. glacialis* C4 and C5. All copepodites stages of *Pseudocalanus* spp. and *C. hyperboreus* were selected against, i.e., captured in lower proportion than their proportion in the plankton (Fig. 7).

### Prey size, prey number and feeding success

**Fig. 8:**
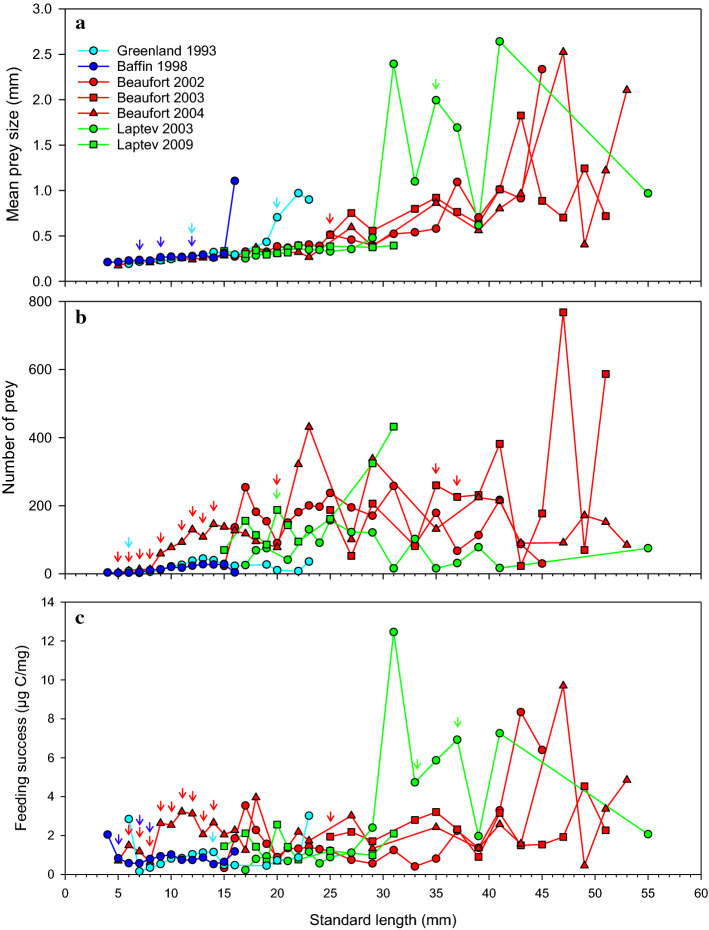
Mean prey size, mean number of prey and mean feeding success by 1-mm (fish 4–25 mm long) and then 2-mm (> 25 mm) length intervals of polar cod sampled in four Arctic seas between 1993 and 2009. Significant differences between two seas are indicated with vertical arrows of the same color as the curve of the sea with the larger value. The sample sizes by standard length intervals are the same as in Fig. 6

Except for a few exceptionally large values in the Greenland Sea and Baffin Bay, mean prey size in larvae 4–25 mm long was similar across the 7 year-sea combinations and increased slowly and regularly with polar cod length (Fig. 8a). Above 25 mm in length, mean prey size in the Beaufort and Laptev Seas (for which data are available) increased more rapidly and became more variable. Despite this variability, mean prey size at length was comparable for the 3 years sampled in the Beaufort Sea (2002, 2003, 2004). In polar cod 31 to 37 mm, mean prey size was significantly larger in the Laptev Sea than in the Beaufort Sea (Fig. 8a). The mean number of prey ingested by polar cod of a given length was often higher in the Beaufort Sea than elsewhere (Fig. 8b). The feeding success of larvae < 15 mm was generally higher in the Beaufort Sea than in the Greenland Sea and Baffin Bay thanks to the ingestion of more prey rather than larger prey (Fig. 8c). In the interval 15–30 mm, the size and number of prey and feeding success differed little between the Beaufort and Laptev seas. The feeding success of juveniles 30–40 mm was generally higher in the Laptev Sea than the Beaufort Sea thanks to larger prey.

### *Calanus glacialis* copepodites and the feeding success of polar cod

**Fig. 9:**
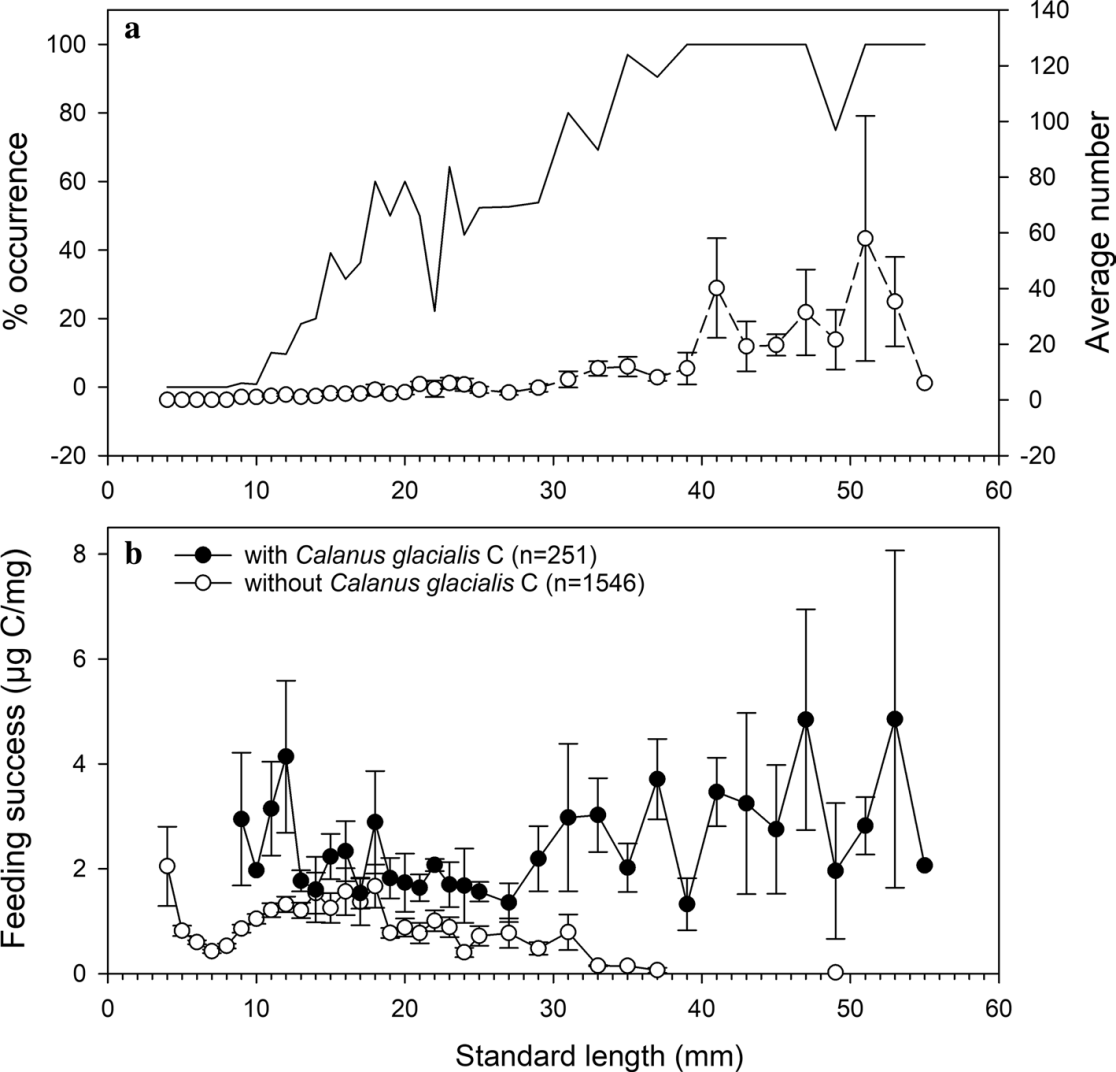
Percent occurrence (line) and average number (circles) of *Calanus glacialis* copepodites in the digestive tract of polar cod (**a**); mean feeding success of polar cod with (full circles) and without (open circles) *C. glacialis* copepodites in the gut (**b**) by 1-mm (fish 4–25 mm long) and then 2-mm (> 25 mm) standard length intervals. The sample sizes by standard length intervals are the same as in Fig. 6. Vertical bars indicate standard errors

The smallest polar cod larva having ingested a *C. glacialis* copepodite was 9.6 mm long. Above that length, the percentage of polar cod capturing *C. glacialis* copepodites increased with length to reach 100% in juveniles > 39 mm long (Fig. 9a). The negative binomial regression showed that polar cod standard length accounted for a significant amount of variance in the number of *C. glacialis* copepodites ingested (*p* < 0.0001, Nagelkerke pseudo-*R*^2^ = 0.74). The average number of *C. glacialis* copepodites per gut was relatively low for polar cod between 10 and 30 mm long, and highly variable for juveniles > 30 mm, with individual numbers of *C. glacialis* copepodites ingested ranging from 0 to 149 (Fig. 9a).

The feeding success of larvae < 12 mm that included *C. glacialis* copepodite(s) in their gut content was ~ 2–4 times higher than that of other larvae of the same size (Fig. 9b). In the range 13–18 mm, feeding success was nearly the same for larvae with or without *C. glacialis* copepodite prey. Above 18 mm, the feeding success of polar cod without *C. glacialis* copepodite prey decreased to zero in fish > 32 mm (Fig. 9b).

## Discussion

### *Calanus glacialis*, the staple food of young epipelagic polar cod in offshore Arctic seas

Earlier studies reported on the numerical importance of copepod eggs and nauplii as prey of polar cod larvae < 14 mm (Drolet et al. 1991; Gilbert et al. 1992; Michaud et al. 1996; Walkusz et al. 2011). Microalgae, rotifers, tintinnids, invertebrate larvae, and appendicularians may also be numerically important in the first weeks of life, especially around river plumes (Gilbert et al. 1992; Walkusz et al. 2011), but are unlikely to contribute substantially to carbon intake due to their diminutive size. In the Greenland Sea, polar cod > 10 mm successfully captured all copepodite stages of small cyclopoids (*Oithona similis, Triconia borealis*) and the C1–C3 of the larger calanoids (*Pseudocalanus* spp., *C. hyperboreus*, *C. glacialis*, *C. finmarchicus*) with positive selection restricted to *C. glacialis* only (Michaud et al. 1996). In the shallow coastal Canadian Beaufort Sea where *C. hyperboreus* is rare, larvae at the flexion stage (15–25 mm) and post-flexion stage (16–34 mm) preyed primarily on the copepodites of *Pseudocalanus* spp. and *C. glacialis*, with *C. glacialis* becoming the main prey (36%) of juvenile fish numerically (Walkusz et al. 2011).

In the present study, the contribution of a given prey taxon to the diet of young polar cod was expressed as carbon intake which accounts for both the number and energy value of the prey taxa. Following Daase et al. (2013) and Ota et al. (2008) we assumed that *Calanus* spp. nauplii found in the gut of polar cod larvae sampled from the end of April to the end of December were *C. glacialis* rather than those of the winter-spawning *C. hyperboreus* whose nauplii tend to emerge earlier in winter and early spring. However, in the productive Barents Sea, *C. hyperboreus* may exhibit a second spawning bout in summer (Melle and Skjoldal 1998). Hence, the possibility remains that in some seas *C. hyperboreus* contributed some of the naupliar carbon assigned to *C. glacialis*.

With this caveat in mind, *C*. *glacialis* eggs, nauplii and copepodites stand out as the main source of carbon for polar cod larvae and juveniles 4 to 56 mm in length (Fig. 6). That the naupliar and copepodite stages were positively selected in the plankton (Fig. 7) suggest some behavioral or mechanical cause to this preference for *C. glacialis*. One possibility is a Goldilocks-type match between the mouth gape of polar cod and the width of *C. glacialis* as both predator and prey grow over the spring–summer months, with *Pseudocalanus* being too small and less rewarding in carbon and *C. hyperboreus* being too large to easily ingest. This level of dependence on a single preferred prey could explain, in part, the long hatching season of polar cod (January to early July; Bouchard and Fortier 2008, 2011). A long hatching season reduces the probability of a recruitment failure due to a temporal mismatch between polar cod and their prey by guaranteeing that the development of at least some cohorts of young fish is synchronous with the development of *C. glacialis*.

In year-sea combinations sampled in late September to December, such as the Beaufort Sea in 2002 and 2003, the copepodites of the small neritic *Pseudocalanus* spp. contributed a significant fraction of carbon intake (Fig. 5). This indicates that polar cod > 15 mm shifted to *Pseudocalanus* spp., a permanent resident of the epipelagic layer, once *C. glacialis* copepodites completed their migration to overwintering depths in mid- to late summer (Madsen et al. 2001; Darnis and Fortier 2014). Hence, while the nauplii of the genus *Pseudocalanus* provided some carbon to polar cod during the pivotal spring–summer first feeding and pre-metamorphosis growth season (Fig. 4), copepodites became significant prey mostly after metamorphosis in late summer, perhaps as a substitute for the disappearing *C. glacialis* copepodites.

As their predatory skills and mouth gape increased after metamorphosis, polar cod > 35 mm infrequently (31% of juveniles) captured C3-F *C. hyperboreus* (Fig. 4), which then contributed a large fraction of total carbon given their large biomass in summer (e.g., C3 = 147 µg C, Forest et al. 2011) compared to *C. glacialis* (C3 = 19.3 µg C, Forest et al. 2011) or the diminutive *Pseudocalanus* (C3 = 0.91 µg C, Liu and Hopcroft 2008). By this size, surviving polar cod are well beyond the critical stages of first feeding and pre-metamorphosis growth during which mortality rates are high (Fortier et al. 2006). Thus, the capture of a large and lipid-rich copepodite of *C. hyperboreus* is likely a welcome bonus, but not necessarily a condition for survival.

Recent studies have established that an early sea-ice break-up and the associated higher spring–summer production of microalgae and zooplankton increase the biomass of juvenile polar cod in August and September in the Canadian Arctic (Bouchard et al. 2017; LeBlanc et al. 2019). The asymptotic relationship between juvenile polar cod biomass and mesozooplankton biomass at the end of summer (LeBlanc et al. 2019) suggests a type II functional response of polar cod juveniles to the abundance of their zooplankton prey (Holling 1959). In these studies (Bouchard et al. 2017; LeBlanc et al. 2019), mesozooplankton biomass was estimated by acoustics and represents the overall Arctic assemblage dominated by copepods (e.g., Ashjian et al. 1997; Kosobokova et al. 1998; Darnis et al. 2008). Our meta-analysis clearly points to *C. glacialis*, *C. hyperboreus* and *Pseudocalanus* spp. as the three calanoid taxa likely underpinning the dependence of juvenile polar cod recruitment on zooplankton production.

### *Calanus glacialis* and the feeding success of young polar cod

A literature synthesis of the feeding ecology of larval fish has revealed several latitudinal patterns (Llopiz 2013). In contrast to low latitudes, young fish at high latitudes tend, during their ontogeny, to (1) capture increasingly large prey; (2) change the composition of their diet; (3) prey on nauplii and then on calanoid copepods with cyclopoid copepods being rare prey; and (4) specialize on a few dominant prey taxa. The feeding characteristics reported here conform to these patterns with developing polar cod feeding on increasingly larger prey and shifting from nauplii to the copepodites of three dominant calanoid prey with a strong preference for *C. glacialis*.

*Calanus glacialis* became increasingly important as the preferred prey and main source of carbon during the early ontogeny of polar cod. In polar cod > 10 mm, the capture of one or more *C. glacialis* copepodites was associated with a higher feeding success (Fig. 9b). Polar cod 13–18 mm nearly compensated for the carbon shortfall of not capturing *C. glacialis* copepodites with other prey. Beyond 18 mm however, the feeding success of polar cod with and without *C. glacialis* copepodite prey increasingly diverged, tending to zero in fish > 32 mm with no *C. glacialis* prey (Fig. 9b). A first but unlikely interpretation of this pattern is that polar cod > 18 mm become increasingly stenophagous, and fish unable to capture *C. glacialis* copepodites are headed for starvation (feeding success = 0). A more likely explanation is that increasingly stenophagous polar cod > 18 mm hunting for *C. glacialis* copepodites increasingly forgo other prey with increasing length. The declining feeding success of fish without *C. glacialis* prey would then reflect the increasing capacity of growing polar cod to withstand fasting while waiting for their preferred prey.

### The dependence of polar cod on *Calanus glacialis* in warming Arctic seas

All indications are that *C. glacialis* is the preferred prey and main carbon source of polar cod during the larval and early juvenile life in the plankton of High Arctic seas where *C. finmarchicus*, when present, is a non-reproducing expatriate (e.g., Melle and Skjoldal 1998; Hirche and Kosobokova 2007). This central conclusion of our study may not necessarily hold for Arctic and sub-Arctic seas and fjords influenced by Atlantic waters where *C. glacialis* and *C. finmarchicus* are sympatric and where the latter reproduces and often dominates the *Calanus* guild. As a first approximation based on the molecular differentiation of *Calanus* spp., such regions include the Kara, Barents, Irminger, and Labrador seas, and some fjords in Svalbard and Norway, but not the White Sea ( Parent et al. 2011; Choquet et al. 2017). In the Kara Sea, *C. finmarchicus* (identified by size) made up 47% by weight of the food intake of 16 polar cod 30–70 mm TL (Prokopchuk 2017). In these and other seas such as Hudson Bay where, to our knowledge, the *glacialis/finmarchicus* conundrum has not been resolved by genetic analyses, the eggs, nauplii and copepodite stages of reproducing indigenous *C. finmarchicus* could contribute to the carbon uptake of young polar cod. Assessing the relative importance of the two species as carbon sources for polar cod in these regions would require the molecular identification of the eggs, nauplii and copepodites of *Calanus* prey. Similar taxonomical uncertainty may arise in the Bering Sea between *C. glacialis* and *C. marshallae* (Frost 1974; Plourde et al. 2005). Furthermore, our conclusion may no longer hold for the seas sampled more than two decades ago (Greenland Sea and Baffin Bay) if these regions have since experienced major shifts in *Calanus* communities.

As the Arctic Ocean and its ancillary seas warm, modeling studies forecast that both *C. glacialis* and *C. finmarchicus* populations will shift poleward (Reygondeau and Beaugrand 2010; Beaugrand et al. 2013; Feng et al. 2018). Thanks to higher temperatures and a longer growth season, young polar cod could benefit from an increased abundance of their preferred prey *C. glacialis* in the northern parts of the Beaufort, Chukchi, East Siberian, Laptev, and Kara Seas, the northern part of the Baffin Bay and the Canadian Archipelago, but less so in the southern part of the Baffin Bay and in the Barents, Greenland, Iceland and Norwegian Seas (Feng et al. 2018). In the southern reaches of the distribution of polar cod, the lipid-rich *C. glacialis* (65% lipids; Falk-Petersen et al. 2009) is increasingly being replaced by the smaller, less lipid-rich (35% lipids; Falk-Petersen et al. 2009) *C. finmarchicus* (e.g., Aarflot et al. 2018; Møller and Nielsen 2019). While prey number determined the feeding success of polar cod larvae < 15 mm, prey size and the capture of large *C. glacialis* copepodites dictated feeding success in juveniles > 30 mm. The pre-winter accumulation of sufficient lipids by preying on large lipid-rich prey is likely a key determinant of the overwinter survival of age-0 polar cod (Fortier et al. 2006; Bouchard and Fortier 2011; Bouchard et al. 2017; Koenker et al. 2018). The impacts of the replacement of *C. glacialis* by the smaller and less energy-rich *C. finmarchicus* as the main source of carbon during the critical early development of polar cod in the plankton remain to be assessed. Indications are, however, that such an imposed shift in diet would be detrimental and could hasten the borealization of Arctic ecosystems by contributing to the replacement of the specialized polar cod as the dominant forage fish of Arctic seas by boreal generalists such as the capelin *Mallotus villosus* and the sand lance *Ammodytes* spp. (e.g., Ingvaldsen and Gjøsæter 2013; Falardeau et al. 2017).

## Data Availability

Data from the study are available in the Polar Data Catalogue (https://doi.org/10.21963/13131).
